# Advanced Safety and Genetic Stability in Mice of a Novel DNA-Launched Venezuelan Equine Encephalitis Virus Vaccine with Rearranged Structural Genes

**DOI:** 10.3390/vaccines8010114

**Published:** 2020-03-02

**Authors:** Dylan M. Johnson, Kevin J. Sokoloski, Jenny D. Jokinen, Tia L. Pfeffer, Yong-Kyu Chu, Robert S. Adcock, Donghoon Chung, Irina Tretyakova, Peter Pushko, Igor S. Lukashevich

**Affiliations:** 1 Department of Microbiology and Immunology, School of Medicine, University of Louisville, Louisville, KY 40202, USA; kevin.sokoloski@louisville.edu (K.J.S.); hoon.chung@louisville.edu (D.C.); 2 Center for Predictive Medicine, School of Medicine, University of Louisville, Louisville, KY 40202, USA; tia.pfeffer@louisville.edu (T.L.P.); yongkyu.chu@louisville.edu (Y.-K.C.); scott.adcock@louisville.edu (R.S.A.); 3 Department of Pharmacology and Toxicology, School of Medicine, University of Louisville, Louisville, KY 40202, USA; jenny.jokinen@louisville.edu; 4 Medigen, Inc., Frederick, MD 21701, USA; itretyakova@medigen-usa.com (I.T.); ppushko@medigen-usa.com (P.P.)

**Keywords:** Venezuelan equine encephalitis virus, DNA vaccine, live-attenuated vaccine, gene rearrangement, genetic stability, reversion, safety

## Abstract

The safety and genetic stability of V4020, a novel Venezuelan Equine Encephalitis Virus (VEEV) vaccine based on the investigational VEEV TC-83 strain, was evaluated in mice. V4020 was generated from infectious DNA, contains a stabilizing mutation in the E2-120 glycoprotein, and includes rearrangement of structural genes. After intracranial inoculation (IC), replication of V4020 was more attenuated than TC-83, as documented by low clinical scores, inflammation, viral load in brain, and earlier viral clearance. During the first 9 days post-inoculation (DPI), genes involved in inflammation, cytokine signaling, adaptive immune responses, and apoptosis were upregulated in both groups. However, the magnitude of upregulation was greater in TC-83 than V4020 mice, and this pattern persisted till 13 DPI, while V4020 gene expression profiles declined to mock-infected levels. In addition, genetic markers of macrophages, DCs, and microglia were strongly upregulated in TC-83 mice. During five serial passages in the brain, less severe clinical manifestations and a lower viral load were observed in V4020 mice and all animals survived. In contrast, 13.3% of mice met euthanasia criteria during the passages in TC-83 group. At 2 DPI, RNA-Seq analysis of brain tissues revealed that V4020 mice had lower rates of mutations throughout five passages. A higher synonymous mutation ratio was observed in the nsP4 (RdRP) gene of TC-83 compared to V4020 mice. At 2 DPI, both viruses induced different expression profiles of host genes involved in neuro-regeneration. Taken together, these results provide evidence for the improved safety and genetic stability of the experimental V4020 VEEV vaccine in a murine model.

## 1. Introduction

Venezuelan Equine Encephalitis Virus (VEEV) is member of the genus Alphavirus in the family *Togaviridae*. There are six antigenic subtypes and seven varieties in the VEEV complex, most of which are endemic to Central and South America [1,2]. Types IAB and IC are associated with epizootic outbreaks in equines, when the virus breaks from its sylvatic cycle between the mosquito vector and rodents or birds [1]. In equines, VEEV presents as a biphasic illness with high fever followed by neurologic disease, resulting in 50%–89% mortality [3]. During infection, equines have a high titer viremia, which can lead to subsequent transmission through the mosquito vector to other equines or humans [3]. However, person-to-person transmission has not been reported. In humans, VEEV causes a biphasic febrile illness followed by myeloencephalitis with high morbidity, including neurological complications, and a mortality rate of approximately 2% [4]. Significant overlap occurs between regions of VEEV endemicity and that of some other tropical disease, which complicates clinical diagnosis. In particular, it has been reported that overlap with areas of dengue virus infection may result in an underestimation of VEEV prevalence [1]. In a population-based study, the seroprevalence of VEEV has been reported to be as high as 75% in some endemic regions [5]. Climate change may be contributing to the increased geographical distribution of *Culex* mosquitos, increasing the population of humans and equine at risk for contracting VEEV [6,7,8].

VEEV was weaponized in the US and former USSR before biological warfare programs were discontinued [4,9,10,11]. Still, VEEV is recognized as a potential biological weapon threat. In the US, VEEV subtypes IAB and IC are regulated under the HHS/USDA Select Agent program due to the severe threat posed to human and animal health, especially in the absence of FDA-licensed human vaccines and therapeutics [12]. Due to the minimum infectious aerosol dose required for infection, VEEV also has a long history of being implicated in laboratory acquired infections [13,14,15].

The first effort to create a VEEV vaccine used a formalin inactivated wild-type virus. However, these experimental vaccines had low immunogenicity and were prone to incomplete inactivation [16]. A live attenuated vaccine, TC-83, was developed by serially passaging VEEV TrD through guinea pig heart cells 83 times while monitoring virulence by intracranial (IC) injection in mice [17]. VEEV TC-83 contains 10 point mutations compared to the VEEV TrD strain, however the attenuation is attributed to two key differences: G to A mutation at position 3 of the 5′ untranslated region, which reduces translation efficiency by restoring Ifit1 restriction, and a threonine to arginine substitution at position 120 of the E2 glycoprotein [18,19,20].

Murine infection with the Trinidad-Donkey strain (TrD) of VEEV has been identified as a good challenge model for vaccine development, in line with the FDA animal rule [21], in addition to the aerosolized model of infection in Rhesus macaques. TC-83 has been tested in human phase I and II trials and became available for individuals “at risk” through the Special Immunization Program under the supervision of the United States Army Medical Research Institute of Infectious Disease (USAMRIID, Fort Detrick, MD) [22]. TC-83 human trials have revealed high reactogenicity, often leading to symptomatic VEEV infection including febrile illness and headaches in up to 40% of vaccinated individuals [23,24]. Additionally, it has been documented that a single dose of TC-83 is often insufficient to illicit protective humoral responses (neutralizing antibodies), as detected by plaque-reduction neutralization tests (PRNT). Individuals with poor responses are usually further immunized (boosted) with formalin-inactivated TC-83 (called C-84) to enhance PRNT titers following TC-83 vaccination [22]. Experimental V3526, a rationally designed VEEV vaccine containing a mutation in the E2 cleavage site of the structural polyprotein, showed promising results in mice. However, the phase I clinical trial revealed unacceptable adverse effects and development of V3526 was discontinued [25]. Recently, chimeric alphaviruses, either with Sindbis virus, or the insect host-restricted Eilat virus, have been tested as VEEV vaccine platforms [24,26,27,28]. An alternate approach has been proposed to create a novel vaccine by the introduction of an internal ribosome entry site to drive control of VEEV structural proteins or capsid [29,30,31].

The limited number of attenuating mutations in TC-83 and the high error rate of the viral RNA-dependent RNA-polymerase (RdRP) seem to be related to the instability of TC-83 attenuation [26]. Substantial progress in alphavirus molecular virology and VEEV pathogenesis has provided a variety of powerful tools to address the safety and immunogenicity of the VEEV TC-83 vaccine. Recently, we have used several approaches to further improve the VEEV TC-83 vaccine: (i) infectious DNA (iDNA) technology to launch a live-attenuated vaccine in vivo [32]; (ii) rearrangement of alphavirus structural genes; and (iii) genetic stabilization of the TC-83-attenuated phenotype [33]. The novel V4020 experimental vaccine was rationally designed by securing the key TC-83 attenuating mutation (E2-T120A), along with a further improvement of attenuation by rearrangement of TC-83 structural genes [33].

In proof-of-concept studies, V4020 provided complete protection against wild-type VEEV TrD challenge, inducing sterilizing immunity in mice [33]. After a single dose of either subcutaneous live V4020 or electroporation with V4020 iDNA, one month later, BALB/c mice were protected from lethal challenge with VEEV-TrD [33]. Cynomolgus macaques, vaccinated with either a single dose or two doses of V4020, were aviremic following an aerosol challenge with VEEV TrD [34]. Here, we compared the safety and genetic stability of V4020 and TC-83 in viral replication kinetics studies in brain tissues, and then assessed the potential to revert to a pathogenic phenotype during passages of the vaccines in mice. In addition to virology methods, NanoString gene expression profiling and RNA-Seq analysis were applied to track viral genetics and host responses during the replication of both attenuated viruses in brain tissues of experimentally infected mice.

## 2. Materials and Methods

### 2.1. Viruses

VEEV TC-83 was obtained from U.S. Army Medical Research and Materiel Command (Fort Detrick, MD, USA), and amplified once in Vero cells. The pMG4020 plasmid for launching was previously described [33]. VEEV V4020 virus was prepared by electroporation of pMG4020 into Vero cells and the generated virus particles were concentrated by ultracentrifugation [33]. Virus titers were determined by infecting monolayers of Vero cells in 24-well tissue culture plates with serial dilutions of virus stocks for 1 h, followed by methylcellulose overlay. Three days post infection, cells were fixed with paraformaldehyde and stained with crystal violet to count infectious plaques.

### 2.2. Vaccine Replication Kinetics in Brain Tissues

BALB/c mice (6–8 weeks old, female, 24 mice per group) were inoculated IC with 10^5^ plaque forming units (PFU) of either V4020 or TC-83 in a total volume of 20 μL of PBS. To prepare for IC inoculation, mice were shaved, anesthetized with isoflurane in an induction chamber, transferred to a nose cone, had the injection site cleaned with first povidone iodine followed by ethanol, and positioned with their head tilted forward. A 27-guage needle (BD) was inserted through the foramen magnum angled to prevent damage to the brain stem. Following injection, mice were monitored during recovery. Mice that did not recover from the IC procedure or that met euthanasia criteria at 1 DPI (about 7% of all IC treated animals with no apparent association to treatment group) were excluded from analysis.

Mice were weighted daily and assessed for signs of clinical disease on a 16-point scale as previously described [33]. Briefly, observation was based on grooming, adjusted grimace scale score, activity, and neurological responses including tail strength, hind leg strength, abdominal curl reflexes, and righting reflexes. On days 1, 2, 3, 5, 7, 9, 11 and 13-post infection, three mice from each group were sacrificed, and brain tissue was collected. Half of the brain was fixed in neutral buffered formalin for 24 h before standard processing and paraffin embedding for histology. A quarter of the brain was placed in 2 mL screw top tubes with 1 mL TRIzol Reagent (ThermoFisher #15596026), and homogenized with glass beads in a bead beater for isolation of RNA by phenol-chloroform extraction. The remaining brain tissue was homogenized in cell culture media and clarified as described previously [35] for plaque assay.

### 2.3. Sequential IC Passages in Mice

BALB/c mice (6–8 weeks old, female, 10 mice per group) were inoculated IC, and, two days post infection, two mice from each group were sacrificed, brain tissue was collected, homogenized, clarified, aliquoted, and stored at −80 °C, as described above. One aliquot from each mouse was titered by plaque assay. A second aliquot was used to prepare the inoculum for subsequent passages after a single freeze–thaw cycle. Each mouse was inoculated with 10^5^ PFU of virus comprised of 5 × 10^4^ PFU per dose originating from each of two mice from the previous passage. The remaining mice in each group were weighed daily and monitored for clinical signs for 14 days or until they met the euthanasia criteria of 25% weight loss or the loss of ability to obtain food and water for a period of 24 h or greater (e.g., complete loss of righting reflex or hind leg paralysis.) At the time of sacrifice, brain tissue was collected as described above.

### 2.4. In Situ Hybridization and Histology

Formalin-fixed paraffin embedded (FFPE) brain tissue was sectioned on a Leica RM2125 RTS microtome to a thickness of approximately 5 μm and mounted on charged glass slides. Slides were baked to dry. Manual hematoxylin and eosin staining was performed as previously described [36]. In situ hybridization was done using RNAScope HD Assay Brown (#322300) using a probe targeting VEEV nsP3 (#404501, Advanced Cell Diagnostics, Hayward, CA, USA) according to the manufacturer’s directions.

### 2.5. NanoString Array

RNA collected during necropsy from the kinetic study on days 5, 7, 9, 11, and 13 post inoculation was isolated from mouse brain homogenized in TRIzol as described above. RNA was similarly collected from mock (PBS) IC-inoculated mice. Purified RNA was quantified on a NanoDrop 2000 spectrophotometer (ThermoFisher, Wilmington, DE, USA), diluted to 10 ng/μL, and sent to NanoString Technologies (NanoString Technologies, Seattle, WA, USA) for analysis. A total of 100 ng of each sample was hybridized to the nCounter Mouse Neuro-inflammation panel (XT-CSO-MNROI1-12) for 16 h at 65 °C. Hybridized RNA was quantified on a NanoString Prep Station and Digital Analyzer Max System (NanoString Technologies, Seattle, WA, USA) with the manufacturer’s high sensitivity protocol. Additional probes to detect VEEV genes (Table 1) were designed and synthesized by NanoString Technologies (NanoString Technologies, Seattle, WA, USA) based on the TC-83 GenBank reference sequence L01443.1. These probes were additionally included in the neuro-inflammation panel.

### 2.6. RNA-Seq

Brain tissue from mice inoculated with passages one through five (P1-P5) of V4020 and TC-83 were collected two days post inoculation, DNAse I treated (Zymo Research #E1010), and purified with an RNeasy mini kit (Qiagen #74104.) Purified RNA was quantified on a NanoDrop 2000 spectrophotometer (ThermoFisher, Wilmington, DE, USA) Samples were prepared for sequencing by rRNA depletion using the NEBNext rRNA Depletion Kit (#E6310) followed by library prep using NEBNEXT Ultra II RNA Library Prep with Sample Purification Beads (#E7775, NEB, Ipswich, MA, USA). Libraries were quality checked on an Agilent BioAnalyzer before sequencing on an Illumina NextSeq 500 with a NextSeq 500/550 High Output Kit v2.5 (75 Cycles) kit (#20024906, Illumina, San Diego, CA, USA).

The sequencing files were imported into Galaxy [37], trimmed using trimmomatic version 0.36.6 [38], aligned to viral reference sequences with BowTie 2 version 2.3.4.2 [39], and a pile-up was generated, filtered and exported to Excel for statistical analysis. Sequences not aligning with the viral references were aligned with the mouse mm10 genome (GenBank assembly accession GCA_000001635.8) using HiSat2 version 2.1.0+galaxy4 [40]. Mouse transcripts were quantified with htseq-count version 0.9.1 [41] and screened for differential gene expression by DESeq2 version 2.11.40.2 [42]. Gene ontologies relating to differential gene expression were detected by goseq mapping software version 1.26.0 [43].

### 2.7. Statistical Analysis

Results showing weights, clinical scores, or viral plaque assay titers are presented as mean ± SEM using the GraphPad Prism version 7 for Windows package (GraphPad Software, LaJolla, CA, USA). Prism was also used for statistical significance testing, as indicated in the applicable figure legends, and to visualize data from NanoString and RNA-Seq experiments as heat-maps.

NanoString nSolver software (NanoString Technologies, Seattle, WA, USA) was used to generate all presented volcano plots and the data presented in supplemental Tables S1 and S3. nSolver also calculated relative cell marker expression. Differences in cell marker expression were tested for significance in Prism by two-way ANOVA followed by Fisher’s LSD post-hoc analysis.

NanoString nSolver Advanced Analysis 2.0 software was used to calculate pathway scores. Pathway scores are calculated based on the first principal component of each measured gene in the KEGG pathway. These differences are then quantified based on the normalized expression of all genes belonging to the pathway. This summarizes the involvement of each tested KEGG pathway for each biological replicate relative to all samples included in the analysis. Pathway scores summarize whether the overall gene expression in a pathway is up- or down-regulated. Values for pathway score were exported from nSolver, normalized to mock infected controls, and tested for significance in GraphPad Prism using a two-way ANOVA with Dunnett’s post-hoc comparison to the mock IC treated group.

NanoString nSolver Advanced Analysis 2.0 software also calculates a “Global Significance Score” (GSS) for a group of samples by measuring the overall differential expression of all genes belonging to a particular KEGG pathway, while ignoring whether each gene is up- or down-regulated. GSS is different from pathway score because it summarizes how much difference there is in pathway involvement between groups. GSS allows for the easier comparison of many pathways between groups, but does not provide meaningful data about the magnitude of overall gene expression.

### 2.8. Ethics Statement

All animal experiments were conducted under protocols approved by the University of Louisville institutional animal care and use committee. Work with live VEEV was conducted at the National Institutes of Health Regional Biocontainment Laboratory on the University of Louisville campus and was done with University of Louisville institutional biosafety committee approval. TC-83 and V4020 were handled with BSL2/ABSL2 practices and are exempt from US federal select agent regulations. Passaging of V4020 and TC-83 in mouse brains was done at ABSL3.

## 3. Results

### 3.1. Kinetics Study: Attenuated Replication of V4020 in Brains of Infected Mice

**Figure 1:**
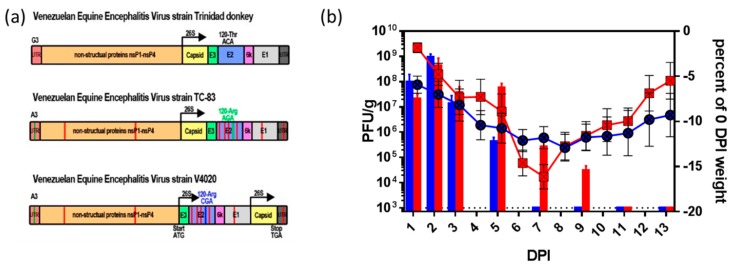
(**a**) Schematic diagram of the genomic structure of VEEV strains TrD, TC-83, and V4020. Red vertical lines denote nucleotide variants (NVs) with a differing sequence from the TrD wild-type strain, which are not thought to play a key role in attenuation. Green vertical lines denote NVs with a differing sequence between TrD and TC-83, which do play a key role in attenuation. A blue vertical line is used to denote the stabilized E2 120Arg NV in V4020. (**b**) Replication kinetics of V4020 and TC-83 in the brain of BALB/c mice (*n* = 24 per group) IC inoculated with 1 × 10^5^ PFU of either V4020 or TC-83 and monitored daily for 13 days. Weight loss (VEEV TC-83 in red square symbols and VEEV V4020 in blue circle symbols.) On days 1, 2, 3, 5, 7, 9, 11, and 13 post inoculation, three mice per time point from each group were necropsied and brain tissue was prepared for VEEV infectious titration by plaque assay to assess viral load in brain tissues (VEEV TC-83 in red bars and VEEV V4020 in blue bars plotted against the left Y axis).

To assess replication kinetics in brain tissues, two groups of BALB/c mice were inoculated with 1 × 10^5^ PFU of either V4020 or TC-83 via the IC route. At 1–2 day intervals, three mice from each group were euthanized and brain samples were taken to determine infectious viral load by plaque titration. Mice from both experimental groups showed symptoms of disease and lost weight (Figure 1b), while a control group of mock-infected mice did not show clinical scores during the observation period (Figure S1). We previously documented that higher clinical scores correlated with weight loss in this model [33]. Direct comparison of percent weight loss to viral load reveals key differences between V4020 and TC-83 at 7 DPI (Figure 1b). Replication kinetics of both attenuated viruses showed similar patters during the first 3 DPI, reaching peak titers of 10^9^ PFU per gram of brain tissue at 2 DPI. After 3 DPI, V4020 replication rapidly declined, with a significant decrease in viral titer at 5 DPI. No infectious V4020 viruses were detected at 7 DPI or at later time points. Conversely, infectious TC-83 was detected at 7 DPI and 9 DPI (with titers of 10^5^ and 10^4^ PFU/g respectively). The high viral loads in TC-83-infected mice seemed to drive more aggressive weight loss than was observed in V4020-inoculated mice (Figure 1b).

**Figure 2:**
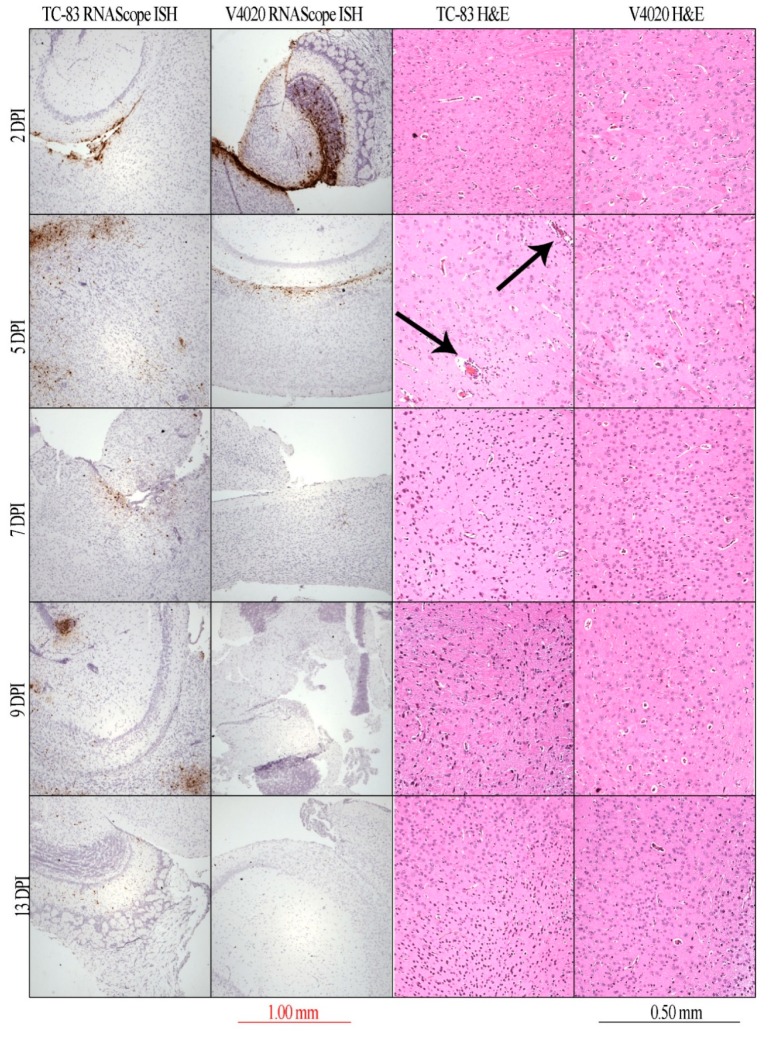
In situ *hybridization* by RNAScope and H&E histology of brain sections. Stained tissue sections of V4020 and TC-83 brain tissue on day 2, 5, 7, 9, and 13 post IC inoculation. Brown staining in RNAScope images indicates the presence of viral RNA detected by an nsP3 in situ hybridization probe. Representative images at 50× magnification for RNAScope and 100× for H&E are shown. Arrows are used to indicate perivascular cuffing.

In line with infectious viral load results, RNAScope in situ hybridization detected strong and extensive signals in the brain tissues of both groups of mice at early stage of the infection (Figure 2, 2 DPI, brown staining). In V4020-infected mice, these signals declined at 5 DPI and were not detectable at later time points. In contrast, in TC-83-infected mice, strong positive hybridization signals remained in brain tissues of TC-83 mice until 9 DPI and were still detectable as late as 13 DPI (Figure 2). Histology (H&E staining) did not reveal clear differences between two groups of mice. Nevertheless, in TC-83-infected mice at 5 DPI, there was a notable increase in perivascular cuffing, a key indicator of VEEV brain inflammation (Figure 2, H&E panels, arrowed). In summary, the replication kinetics study documented a more attenuated profile of VEEV V4020 replication in comparison with the VEEV TC-83 experimental vaccine.

### 3.2. Neuro-Inflammation in Brain Tissues of Mice Infected with V4020 vs. TC-83 Assessed by Direct Multiplexed Measurement of Gene Expression

NanoString nCounter gene expression technology allows direct measurement of mRNA expression levels in tissues without enzymatic reactions [44]. This technology is more sensitive than DNA microarrays and comparable to real-time PCR measurements for individual genes [45,46]. In this study, the commercially available Mouse Neuro-Inflammation color-coded probe panel [47], enabling the quantification of 770 genes involved in 23 KEGG-pathways and processes, was used to assess host cell responses in brain tissues of V4020- and TC-83-infected mice. In addition, customized barcoded VEEV-derived probes were designed and included in this panel to detect and quantify the expression of individual VEEV genes.

**Figure 3:**
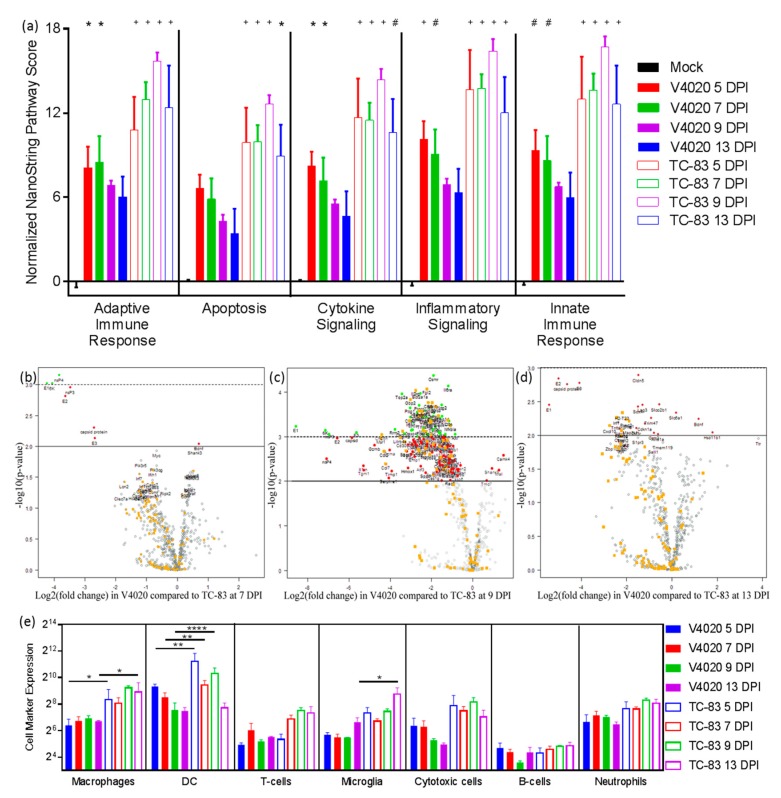
Detection and profiling genes involved in neuro-inflammation pathways. RNA was isolated from the brains of mice inoculated with V4020 or TC-83 and was quantified by NanoString nCounter assay using the mouse neuro-inflammation panel with additional probes to detect VEEV genes. (**a**) NanoString nSolver software was used to calculate a pathway score to summarize the differential expression data from all genes in the pathway based on the first principal component of normalized gene expression. Pathway scores were normalized to the average score for mock IC inoculation. Two-way ANOVA followed by multiple Student’s *t*-tests with Dunnett’s correction were used for post-hoc comparison to mock inoculated controls. * *p* < 0.01, ^#^ *p* <0.001, + *p* < 0.0001. Volcano plot presentation of differential gene expression in V4020-infected mice compared to TC-83-inoculated mice on (**b**) day 7, (**c**) day 9, and (**d**) day 13. Differentially expressed genes color coded according to adjusted significance where genes in red above the solid line represent the threshold of *p* < 0.01 and genes in green are above the dashed line, which represents *p* < 0.001. Genes relating to the inflammatory KEGG pathway are labeled and shown in orange boxes (and are not additionally color-coded by significance, but this can be determined by the lines on the graph). All significantly differentially expressed genes, (or the top 50 overall most significant genes for 7 DPI) have text names. (**e**) Cell type abundance in brain samples was determined by markers present in the NanoString mouse neuro-inflammation panel on days 5, 7, 9, and 13 with significance determined for each time by a two-way ANOVA without matching, followed by Fisher’s least standard difference (LSD) post-hoc analysis. * *p* < 0.05, ** *p* < 0.01, **** *p* < 0.0001.

Pathway scoring is a summary statistic that reflects the relative KEGG pathway involvement based on gene expression. As seen in Figure 3a, inflammatory and cytokine signaling, as well as innate and adaptive immune response pathways, were significantly upregulated in both TC-83 and V4020-inoculated mice compared to mock-inoculated controls at 5 and 7 DPI. However, the magnitude of upregulation is much greater for TC-83 compared to V4020, and remains significantly higher than mock-infected mice at 9 and 13 DPI. Conversely, in V4020-inoclulated mice, pathways scores declined and are no longer statistically different from mock treated animals by 9 DPI (Figure 3a). In general, pathway scores peak at 5 DPI for VEEV V4020 and 9 DPI for VEEV TC-83 (Figure 3a) which corresponds to the last day that infectious viral particles are detected by plaque assay in brain tissue (Figure 1b).

Volcano plots provide visualization of the magnitude of differential gene (host and viral) expression in V4020 treated mice compared to TC-83 (X axis, log2[fold change]), along with statistical significance (Y axis, -log10[*p*-value]). There were no significant differences in gene expression patterns at 5DPI (Figure S2a). At 7, 9 and 13 DPI, VEEV viral genes were downregulated in V4020-inoculated mice compared to TC-83-inoculated animals (Figure 3b–d, named viral genes, red [*p* < 0.01] and green [*p* < 0.001] colored genes). At 9 DPI, the greatest number of genes displayed large log2-fold changes, and the changes were highly statistically significant (Figure 3c). Among these, host genes related to inflammation (labeled by orange boxes) were clearly identified. These inflammation related genes were less abundant at 13 DPI (Figure 3d). All differentially expressed genes, with a greater than 2 log2-fold change in at least one passage, are listed in Table S1. VEEV viral genes E1, 6K, nsP4, nsP3, E2, capsid protein and E3 showed the greatest average magnitude of change, followed by host genes related to inflammation, including interleukin 1 receptor antagonist (Il1rn), granzyme B (Gzmb), macrophage scavenger receptor 1 (Msr1), leukocyte immunoglobulin like receptor B4 (Lilrb4a), granzyme A (Gzma) and chemokine genes (Ccl2, Cxcl10, ccl7, Cxcl9).

A heat-map of GSS scores showed clearly distinguished patterns between V4020 and TC-83 samples (Figure S2b). The GSS scores for mock and V4020 inoculated mice at 9DPI were very similar across all pathways, suggesting the processes involved in neuro-inflammation, damage, repair and adaptive immune responses have returned to baseline values at this time point. Meanwhile, “inflammatory signaling” and “neuron and neurotransmission” GSS had markedly different patterns between V4020 and TC-83 groups (Figure S2c,d, respectively).

NanoString technology can also quantitate predicted cell type abundance based on mRNA expression probes specific for cell type markers presented in the Mouse Neuro-inflammation panel. Markers of macrophages, DCs, and microglia were expressed with statistically significant differences in brain tissues of V4020 mice in comparison with TC-83-inoculated mice. As seen in Figure 3e, in brain tissues of TC-83-inoculated mice, the expression of DC markers was strongly upregulated at 5, 7, and 9 DPI. Macrophage cell markers were generally upregulated in TC-83 compared to V4020, with statistically significant differences at 5 and 13 DPI. In addition to macrophages, markers of microglia were also clearly upregulated in TC-83-inoculated mice. In summary, direct multiplexed measurement of genes involved in mouse neuro-inflammation pathways clearly demonstrated upregulated patterns of genes involved in neuro-inflammation in mice inoculated with VEEV TC-83 versus VEEV V4020.

### 3.3. Clinical Profiling of Mice during Serial Intracranial Passages of V4020 and TC-83

**Figure 4:**
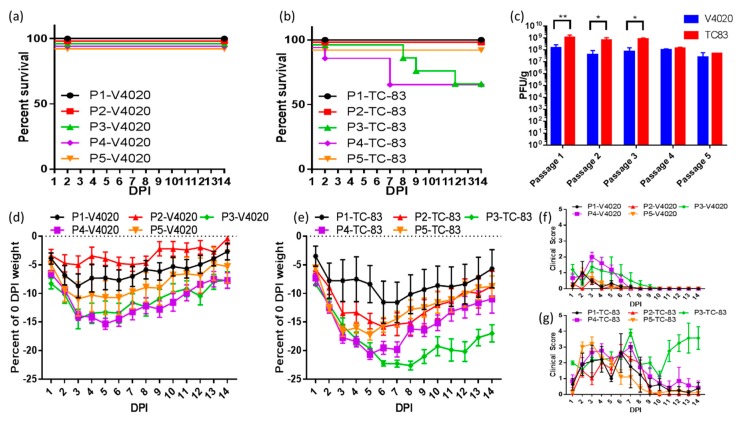
Groups of BALB/c mice (*n* = 10) were inoculated via the intracranial route with 1 × 10^5^ PFU of either V4020 or TC-83. Two days after inoculation, two mice from each group were sacrificed, and their brains were collected, homogenized, and used to make clarified viral stocks for inoculation of a subsequent group. This process was repeated four times for a total of five passages, including the initial inoculation (named in the schematic of PX-Virus, where X is the passage number and virus is either V4020 or TC-83.) Survival of (**a**) V4020 and (**b**) TC-83 passage groups was tracked during this time. (**c**) Brain titers from the mice sacrificed on day 2 post inoculation. Two-way ANOVA followed by Fisher’s LSD post-hoc analysis were used to compare viral titers, * *p* < 0.05, ** *p* < 0.01. Daily weights (**d**,**e**) and clinical scores (**f**,**g**) were taken from the remaining mice challenged with either V4020 (**d**,**f**) or TC-83 (**e**,**g**) daily for 14 days.

Serial IC passages in mice are widely used to assess the phenotypic and genetic stability of alphavirus attenuation. This approach was used to compare the V4020 and TC-83 experimental vaccines. As seen in Figure 4, five sequential IC passages (P1-P5) of V4020 and TC-83 in BALB/c mice resulted in striking differences in clinical profiles. First, throughout the five serial passages, all V4020 treated mice survived, while three out of eight TC-83 mice met euthanasia criteria in the groups which received passage 2 (P2) and P3 virus (Figure 4a,b). Second, measurement of viral load at 2 DPI in brain tissues of mice revealed lower titers during P1-P3 in V4020 mice, and these differences were statistically significant. (Figure 4c). Third, mice infected with V4020 isolated from previous brain passages tended to have weight loss predominantly during the first 3 DPI, then gained weight after this time point. Some mice (e.g., in P1- and P2-V4020 groups) nearly fully recovered their initial weight by the end of observation period (Figure 4d). In contrast, mice in TC-83 groups tended to have a prolonged, more profound, weight loss. No mice in TC-83 groups recovered their initial weight (Figure 4e). Finally, clinical scoring of mice revealed that mouse-brain-passaged V4020 had clinical manifestations peaking at 2-3 DPI. After this time point, clinical signs were ameliorated in all mice, and, after 7-8 DPI, no V4020 animals had scored clinical symptoms (Figure 4f). In great contrast, mouse brain passaged TC-83 caused more severe clinical scores that persisted in some mice at 14 DPI (Figure 4g). Interestingly, clinical symptoms of TC-83 inoculated animals displayed a biphasic pattern (e.g., P3 and P4 TC-83, Figure 4g). These results indicate that IC passages of the VEEV V4020 experimental vaccine did not result in a change in the pathogenic profile from the initial V4020 inoculation (P1). In contrast, VEEV TC-83 passages resulted in the generation of viruses which caused aggravated experimental disease, which resulted in the euthanasia of 13.3% of TC-83 treated mice.

### 3.4. Viral and Host RNA-Seq Profiles of Brain Samples, P1-P5

**Figure 5:**
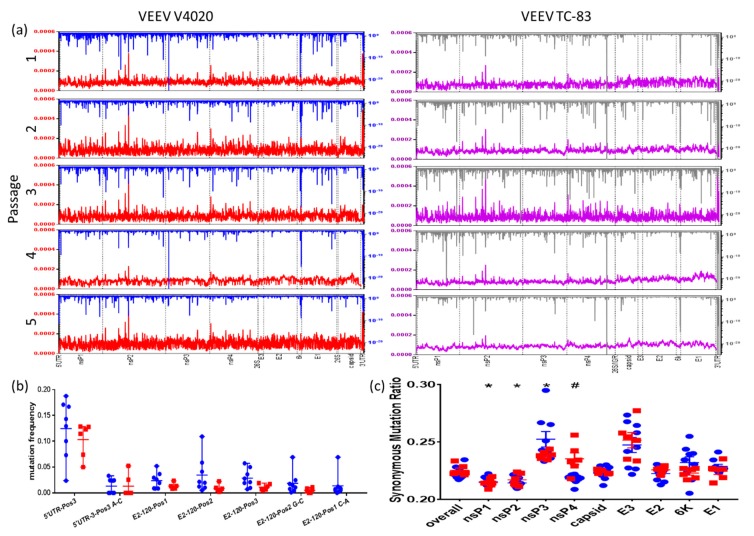
RNA-Seq by Illumina NextSeq was run on rRNA depleted RNA isolated from brain tissue of mice receiving the passaged virus on day 2 post IC inoculation. Sequences were quality trimmed with trimmomatic and aligned to VEEV TC-83 or V4020 reference sequences with BowTie2. Pile-ups were generated and filtered in Galaxy before being exported to excel to calculate normalized read depth (**a**), statistical significance of mutations (**a**), mutation frequency (**b**) and SMR (**c**). (**a**) Normalized read depth plotted on the left y-axis (red for V4020, purple for TC-83) was calculated by dividing the number of virus-aligned reads at each position by the sum of the number of reads for every position. Statistical significance of mutation rate as a Z-score, plotted by nucleotide position on the right y-axis (blue for V4020 and grey for TC-83), was calculated in Excel using the “NORM.DIST” function to calculate a probability density function based on the mean and standard deviation of each biological sample. The boundaries between genes and certain features are marked with a dashed vertical line and labeled at the bottom of the panel. Z-scores over 10^−25^ are reported as this value. Passages 2–5 of V4020 and Passage 1 of TC-83 are the average of two biological replicates. All other data are representative of a single biological replicate. (**b**) Mutation frequency calculated as a ratio of a mutation occurring at the identified location divided by the total number of reads for each reported location for V4020 (blue) and TC-83 (red). (**c**) SMR for the overall coding sequences and individual genes of V4020 (blue) and TC-83 (red). * *p* < 0.05 for the indicated gene compared to the overall SMR by one-way ANOVA with Dunnettt’s post-hoc test, ^#^ *p* < 0.05 for the SMR of nsP4 in V4020 compared to TC-83 by student’s *t*-test. Data in this figure are averaged from two mice per group, except for V4020 passage 1 and TC-83 passages 2 through 5, which represent a single mouse.

During serial passaging in brain, RNA samples from tissue samples collected at 2 DPI were analyzed by Illumina NextSeq 500 to assess genetic variations in V4020 and TC-83 viral populations. In general, as seen in Figure 5a, the V4020 virus had lower rates of mutations that had high statistical significance throughout passages P1-P5 (downward spikes in blue for V4020 or grey for TC-83). Read depth among sequenced samples depicted in red for V4020 or purple for TC-83 was similar (Figure 5a). At 2 DPI, neither virus showed signs of selective pressure driving the reversion of either of the key attenuating mutations: the 5′UTR position 3-A or the E2-120 codon (Figure 5b). The rates of transversion and transition mutations were similar among all samples (Figure S3a,b). Synonymous to non-synonymous mutation ratios (SMR) were significantly lower for nsP1 and nsP2 genes and elevated for nsP3 genes in samples derived from both viruses (Figure 5c). The SMR ratio tended to increase for highly statistically significant mutations (Figure S3c). Interestingly, a significantly higher SMR was observed in the nsP4 (RdRP) gene of TC-83 compared to V4020. In particular, many more nucleotide variants (NVs) were detected in nsP3 and nsP4 of passage 2 and 3 of TC-83 than V4020, with several of these persisting through passages 4 and 5. Fewer NVs were observed in the capsid gene of V4020, with the largest differences in P2, P3, and P4.

Sequence reads mapping to the murine host allowed a basic analysis of the host response at 2DPI for V4020 compared to TC-83 infected mice. DESeq2 analysis with two factors, virus (V4020 or TC-83) and passage number, revealed 21 differentially expressed host genes. However, none of these appear to segregate in a clear way to inoculation with V4020 or TC-83 (Figure S4a). A full list of differentially expressed host genes generated by DESeq2 in both the two factor analysis and, for (one factor, virus,) of each passage is presented in Table S2. Based on RNA-Seq at 2 DPI and NanoString analysis from 5-13 DPI, 97 host genes were identified to be differentially expressed between V4020, and TC-83 in both assays (Table S3). Interestingly, among these genes—immune regulators Tbx21 (a key regulator of cytotoxic T-cell response) and Eomes, complement genes C1qa and C1qb, the cytokine Cx3cl1, and the lysosomal marker Lamp2—were identified as being differentially expressed in relation to both virus and time point (Table S3, fold changes in V4020 compared to TC-83). Ontological analysis of differentially expressed genes compiled from each individual passage highlighted that the pathways most likely to be differently regulated in V4020 compared to TC-83 IC-inoculated mice at 2 DPI predominantly involve neurogenesis and brain function (Figure S4b). In summary, RNA-Seq profiling of samples collected at 2 DPI during serial passages revealed lower rates of viral mutations in V4020 samples, and different patterns of host gene expression associated with neurogenesis and brain function in V4020- and TC-83-inoculated mice.

## 4. Discussion

Historically, live attenuated vaccines against viral infections have been among the safest and most efficacious medical interventions, and they continue to be the most cost-effective preventive measures [48,49]. In recent years, live attenuated vaccines against influenza (FluMist), rotavirus (Rotarix) and herpes (Zostavax) infections were licensed for human use. This indicates that the powerful tools of molecular virology which are currently available can be successfully applied to address the inherent weaknesses of these vaccines: genetic stability and reversion to wild-type phenotypes [50]. The TC-83 vaccine for VEEV was developed almost 60 years ago using the “classical” technique of multiple passages in vitro and in vivo, which resulted in two attenuating mutations, the first in the 5′-end untranslated region, position 3 (G > A) and the second in the E2 envelope glycoprotein, position 120 (Thr > Arg) [17]. Since the 1960s, despite numerous attempts to develop better vaccines, TC-83 is the only vaccine in human use under an IND protocol (Special Immunization Program) which has allowed it to be tested in Phase II trial (NCT03531242, NCT00582504, NCT00582088, NCT03051386, https://ClinicalTrials.gov). Nevertheless, TC-83 is reactogenic and poorly immunogenic in ~18%–20% of vaccinees, which raises serious safety concerns.

The small number of attenuating mutations seems be associated with the genetic instability and reactogenicity of TC-83. To address this issue, we have applied several approaches to develop an advanced VEEV vaccine, V4020 [33], including: (i) rearrangement of structural genes, (ii) introduction of an additional sub-genomic promoter (26S) downstream from the glycoprotein genes; (iii) synonymous translational codon replacement to secure the attenuating E2 mutation TC-83; and (iv) the rescue of V4020 from a molecular clone (cDNA) to limit the population heterogeneity of the V4020. Here, we compared the replication kinetics of V4020 and TC-83 in the brain following IC inoculation of mice, and performed five IC mouse passages, using brain tissues as the source of the virus, to assess the genetic stability and heterogeneity of these experimental vaccines.

It is cannot be excluded that incomplete attenuation, characterized by the emergence of viruses with reversions, is responsible for the pathogenic features and adverse effects of TC-83 vaccination [9]. Notably, viruses with reversion mutations have been isolated from throat swabs of individuals vaccinated with TC-83 (Jahrling PB, unpublished data) [51]. Antiviral host responses following inoculation, along with the delay in the virus reaching the CNS, probably have synergistic effects on the attenuation of neuro-virulence. IC inoculation of experimental mice is a useful tool for short-cutting the natural infection route and directly examining the neuro-virulence of live attenuated vaccines.

We have shown here that IC inoculation of VEEV V4020 resulted in a more attenuated pattern of replication compared with TC-83, as determined by clinical manifestations (weight loss) and viral load in brain tissues of BALB/c mice (Figure 1b). The most striking difference between two infections was more than 2 log_10_ differences in infectious viral load (PFU/g) in the brain at 5–7 days after IC inoculation. This was in line with RNAScope in situ hybridization data (Figure 2). High viral load seemed to be the major driving force, resulting in a spike in weight loss (which has previously been shown to correlate with clinical score [33]) at this time point in TC-83-infected mice.

C3H/HeN mice are considered a more susceptible VEE model in comparison to BALB/c mice [52]. However, IC inoculation of BALB/c with wild-type VEEV TrD and C3H/HeN mice with wild-type VEEV TrD or with VEEV TC-83 resulted in the same LD50 value, around 20 PFU [51]. In contrast, the experimental VEEV V3526 vaccine was not lethal in C3H/HeN mice. However, the poor safety profile of this vaccine in Phase I trial prevented further development [53]. The predominant adverse effects of VEEV TC-83 tend to be fever and headache [23], which coincide with the key symptoms of natural human VEEV infection [54]. In non-human primate models, central nervous system (CNS) invasion by TC-83 causes perivascular infiltration, gliosis, and signs of neurological disease [55]. IC inoculation of BALB/c mice recapitulates key features of neuro-virulence following TC-83 vaccination including perivascular cuffing (Figure 2), monocyte accumulation in the CNS (Figure 3e,) and clinical signs of infection (Figure 1b, and Figure 4).

NanoString technology with a custom-made panel of barcoded probes for individual viral genes, combined with a commercially available mouse neuro-inflammation panel, allowed the direct detection and assessment of the involvement of viral and host genes in neuro-inflammation of brain tissues in V4020- and TC-83-inoculated BALB/c mice. Volcano plots clearly illustrated differential expression of the genes in V4020 mice versus TC-83 mice at 5, 7 and 9 DPI, and (among host genes) genes related to inflammation pathways and processes prevailed in TC-83 mice (Figure 3, Table S1). In line with these results, markers of macrophages, DCs, and microglia were strongly upregulated in TC-83-infected mice, suggesting the involvement of these cells in inflammation. As further evidence that immune infiltration into the brain is responsible for weight loss and clinical manifestations (Figure 1), histology revealed perivascular inflammation foci in TC-83 brain H&E-stained sections (Figure 2, H&E staining, arrowed).

Reversion to a neuro-virulent phenotype represents the greatest risk in using a live attenuated vaccine against VEEV [26]. It has been reported that attenuated strains of VEEV can revert to causing neuro-virulence in as few as three serial passages in mouse brain [17,56]. Indeed, in our experiments P3 and P4 TC-83 isolates induced clinically manifested infection and death. Interestingly enough, after serial passages in brain, TC-83 induced a biphasic disease (Figure 4g), a classical description of the murine model of wild-type VEEV [9]. In contrast, after five IC passages, V4020 did not induce lethal disease. Clinical manifestations were mild or moderate and disappeared in most animals within one week of IC inoculation. VEEV pathogenesis seems to be due to complex polygenic traits that are difficult to relate to NVs in the viral genome [10]. Unique NVs were not detected from more neuro-virulent passages of TC-83. However, TC-83 viruses from passages P2 and P3 had a high level of NVs which mapped within nsP4 versus V4020 viruses (Figure 5a). SMR, defined as the total number of base pair mutations without change in the amino acid code divided by the total number of base pair mutations, was used to detect purifying selection as the evolutionary mechanism of VEEV evolution [57]. For both TC-83 and V4020 experimental vaccines, which nearly completely share a sequence identity (except, as previously noted, for V4020 rearrangement and stabilizing E2 position 120 mutation), nsP1 and nsP2 had significantly reduced SMR over five serial passages in mouse brain, suggesting that mutation in these genes is likely deleterious. Conversely, E3 had an increased SMR. While the time was too short (2 DPI) to consider selective pressure and adaptive immunity involvement, it is interesting to note that E3 has been identified as a key target of humoral responses which are protective against VEEV-TrD [58]. It has been reported that TC-83 strains with lower-fidelity RdRP are more attenuated and immunogenic [59]. nsP4, the viral RdRP [10], had a higher SMR in TC-83 than V4020, however we did not detect differences in mutation frequency across the genome (Figure S3d), suggesting that no real difference in RdRP fidelity arose during passaging. 

## 5. Conclusions

The lack of apparent differences in mutation frequency at key attenuating mutations, and the further lack of evidence for any positive selection during the time points studied, suggests that the strategy of stabilizing the E2 T120A is valid to decrease the appearance of revertants which would potentially arise by random mutation in the viral quasi-species at the peak of viral replication following vaccination. Structural rearrangement seems to additionally secure the attenuated phenotype of V4020. Taken together, these results indicate that V4020 is a safer and genetically more stable alternative to TC-83. Successful testing of V4020 in a non-human primate aerosol challenge model provides an additional argument for the clinical development of this experimental vaccine [34].

## Figures and Tables

**Table 1:** NanoString probes used for the detection of Venezuelan Equine Encephalitis Virus (VEEV) genes.

Target	Probe
6K	ACAAT AACCA ACAGA TGTTC TGGAT TCAAT TGCTG ATCCC TCTGG CCGCC TTGAT CGTAG TGACT CGCCT GCTCA GGTGC GTGTG CTGTG TCGTG CCTTT
capsid	ATCGA CAACG ACGTT CTGGC CGCGC TTAAG ACGAA GAAAG CATCC AAATA CGATC TTGAG TATGC AGATG TGCCA CAGAA CATGC GGGCC GATAC ATTCA
E1	CACCA GGGTG TCAGA AACAC CGACA CTTTC AGCGG CCGAA TGCAC TCTTA ACGAG TGCGT GTATT CTTCC GACTT TGGTG GGATC GCCAC GGTCA AGTAC
E2	CTTAA AAGGA AAACT GCATG TCCCA TTCTT GCTGG CAGAC GGCAA ATGCA CCGTG CCTCT AGCAC CAGAA CCTAT GATAA CCTTC GGTTT CAGAT CAGTG
E3	CCACC ATGTG TCTGC TCGCC AATGT GACGT TCCCA TGTGC TCAAC CACCA ATTTG CTACG ACAGA AAACC AGCAG AGACT TTGGC CATGC TCAGC GTTAA
nsP3	GTGTG CTCAT CCTTT CCATT GCCGA AGTAT AGAAT CACTG GTGTG CAGAA GATCC AATGC TCCCA GCCTA TATTG TTCTC ACCGA AAGTG CCTGC GTATA
nsP4	GCTGG TTAGG AGATT AAATG CGGTC CTGCT TCCGA ACATT CATAC ACTGT TTGAT ATGTC GGCTG AAGAC TTTGA CGCTA TTATA GCCGA GCACT TCCAG

## Supplementary Materials

The following are available online at https://www.mdpi.com/2076-393X/8/1/114/s1, Table S1: Differentially expressed genes detected by NanoString, Table S2: Differentially expressed genes for a “virus x passage” RNA-Seq model, Table S3: Differential expression of genes detected by both NanoString and RNA-Seq. Figure S1: A group of BALB/c mice (n = 5) were inoculated IC with 10 μL of normal saline as a control for animals similarly inoculated with virus. These mice were followed for 14 days for (a) weight, (b) clinical score, and (c) survival. Figure S2: RNA was isolated from the brains of mice inoculated with V4020 or TC-83 as described in Figure 3 and was quantified by NanoString nCounter assay using the mouse neuro-inflammation panel with additional probes to detect VEEV RNA. (a) Differential gene expression in V4020 compared to TC-83 inoculated mice on day 5. (b) NanoString nSolver software was used to calculate a GSS (a measure of the overall differential expression of genes belonging to a particular KEGG pathway, ignoring whether each gene is up- or down-regulated) for each experimental grouping. Calculated NanoString GSS of the (c) inflammatory signaling and (d) neurons and neurotransmission gene sets for V4020 (blue) and TC-83 (red) on the indicated days. Figure S3: Frequency of transitions and transversions by base pair, calculated as the counts of the specified mutation occurring divided by the total reads at all positions with the specified reference nucleotide grouped by (a) virus and passage or by (b) virus. (c) The SMR for all positions (overall) with a significance of a Z-score less than the indicated value. (d) Mutation frequency by virus and passage for the whole virus genome from 2 DPI RNA-Seq calculated as the sum of the number of non-reference reads at every position divided by the sum of the total number of reads at every position. Figure S4: RNA-Seq data were generated and trimmed, as described in Figure 5. All analysis described was performed using the named modules in the Galaxy web-based platform. Sequences were aligned to the mouse mm10 genome using HiSat2, and features were counted with htseq-count. (**a**) All genes with significant differential expression in a model accounting for both virus vaccine strain and passage number are presented in a heat map showing fold change. All genes with differential expression with an adjusted *p*-value greater than 0.05 from each individual passage that were detected by DeSEQ2 were used as the input to run GOseq on each passage individually. The pathways reported by GOseq for each passage were combined and sorted by significance. (**b**) The overall top 50 over-represented ontologies are displayed in a heat map depicting the associated adjusted *p*-value associated with the likelihood of over-representation from GOseq. *p*-values less than 10^−10^ are reported as 10^−10^.
