# Supplementary material for: Advanced Safety and Genetic Stability in Mice of a Novel DNA-Launched Venezuelan Equine Encephalitis Virus Vaccine with Rearranged Structural Genes
Source: Vaccines (Basel). 2020 Mar 2;8(1):114. doi: 10.3390/vaccines8010114 (PMC7157698; doi:10.3390/vaccines8010114)
Supplement: Supplementary file 1 [file vaccines-08-00114-s001.zip › sup/vaccines-734347 proof sup .docx]

Supplemental Figures

**
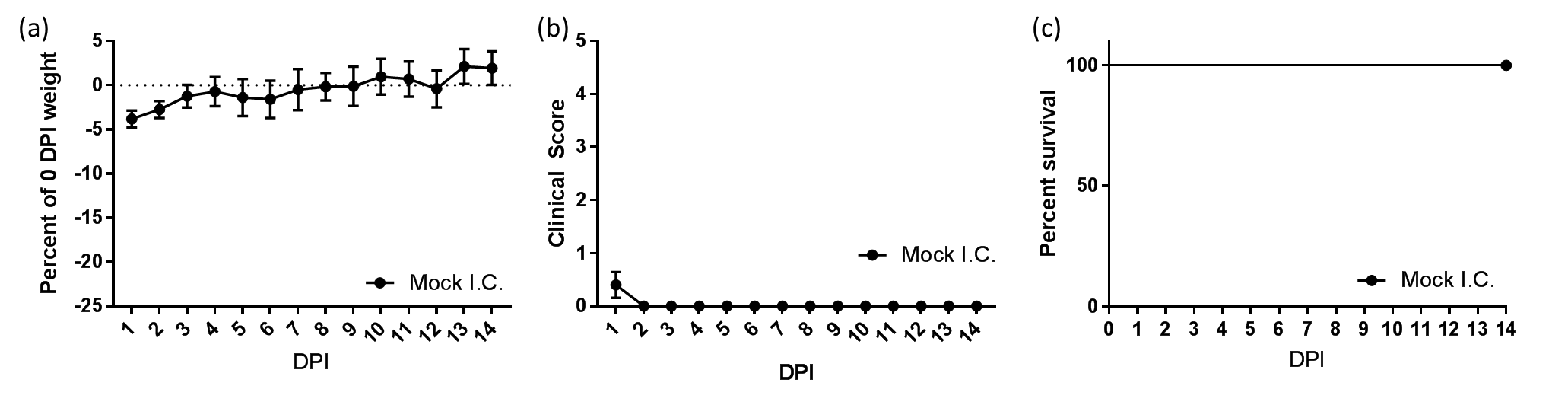
**

**Figure S1.** A group of BALB/c mice (*n* = 5) were inoculated IC with 10 μl of normal saline as a control for animals similarly inoculated with virus. These mice were followed for 14 days for (**a**) weight, (**b**) clinical score, and (**c**) survival.


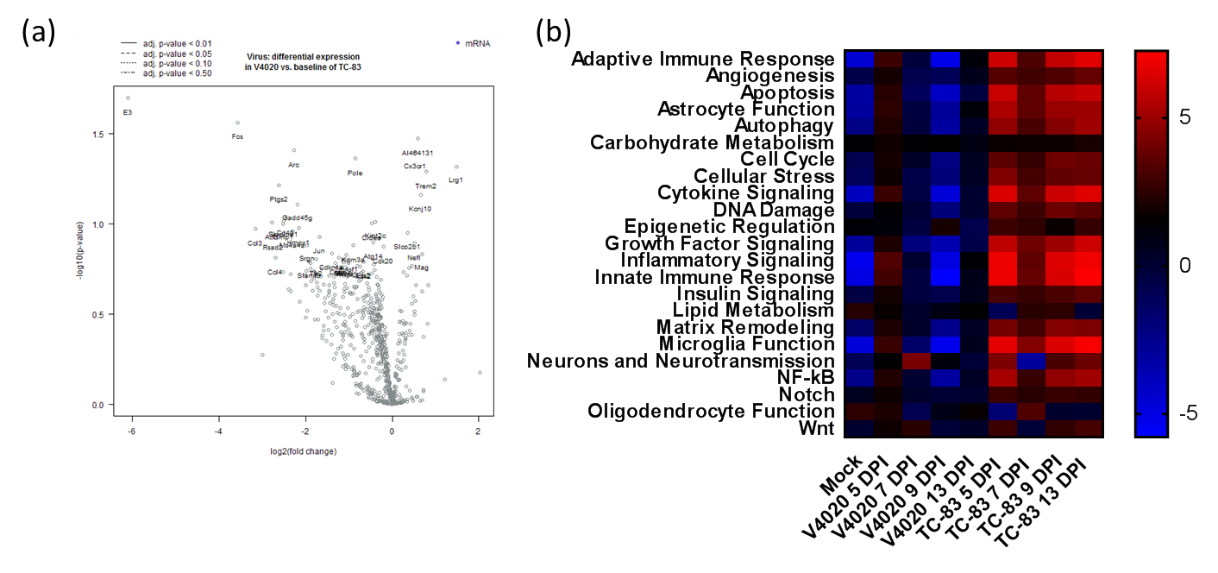

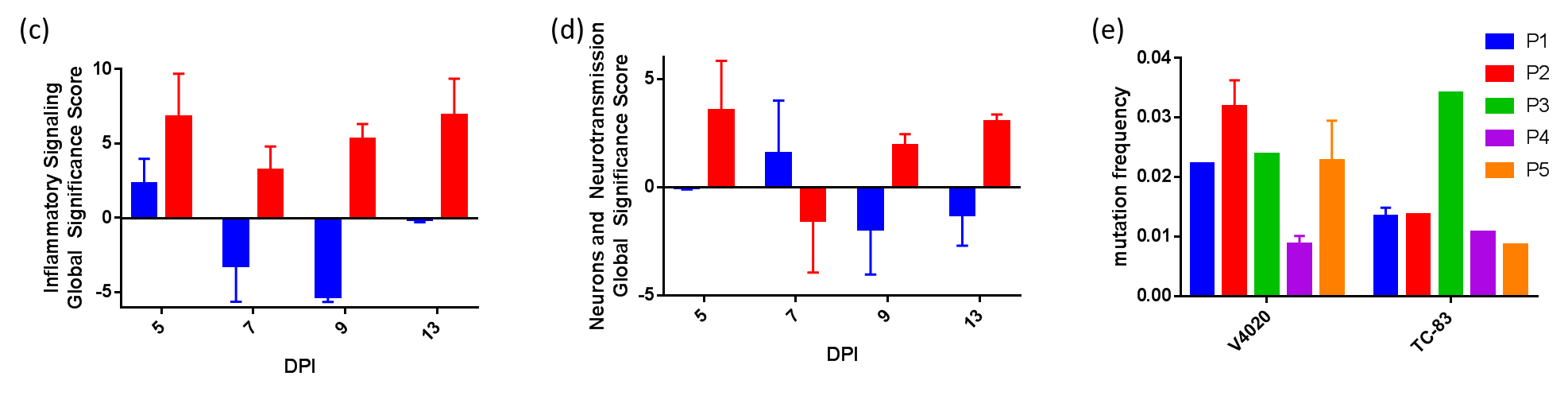


**Figure S2.** RNA was isolated from the brains of mice inoculated with V4020 or TC-83 as described in Figure 3 and was quantified by NanoString nCounter assay using the mouse neuro-inflammation panel with additional probes to detect VEEV RNA. (**a**) Differential gene expression in V4020 compared to TC-83 inoculated mice on day 5. (**b**) NanoString nSolver software was used to calculate a GSS (a measure of the overall differential expression of genes belonging to a particular KEGG pathway ignoring whether each gene is up- or down-regulated) for each experimental grouping. Calculated NanoString GSS of the (**c**) inflammatory signaling and (**d**) neurons and neurotransmission gene sets for V4020 (blue) and TC-83 (red) on the indicated days.


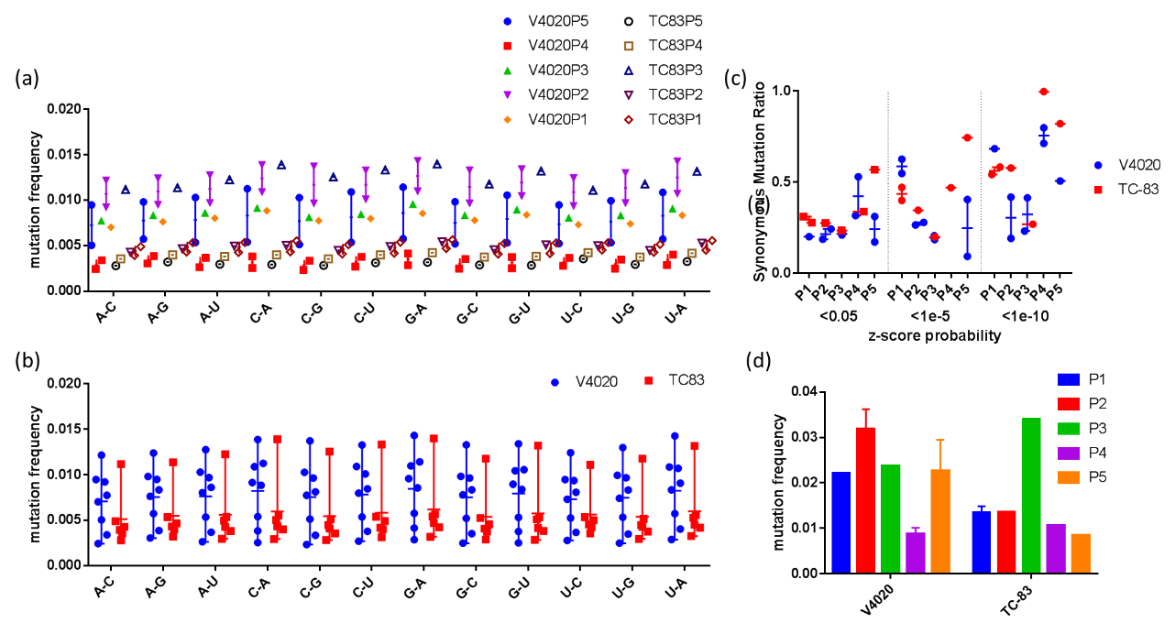


**Figure S3.** Frequency of transitions and transversions by base pair calculated as the counts of the specified mutation occurring divided by the total reads at all positions with the specified reference nucleotide grouped by (**a**) virus and passage or by (**b**) virus. (**c**) The SMR for all positions (overall) with a significance of Z-score less than the indicated value. (**d**) Mutation frequency by virus and passage for the whole virus genome from 2 DPI RNA-Seq calculated as the sum of the number of non-reference reads at every position divided by the sum of the total number of reads at every position.


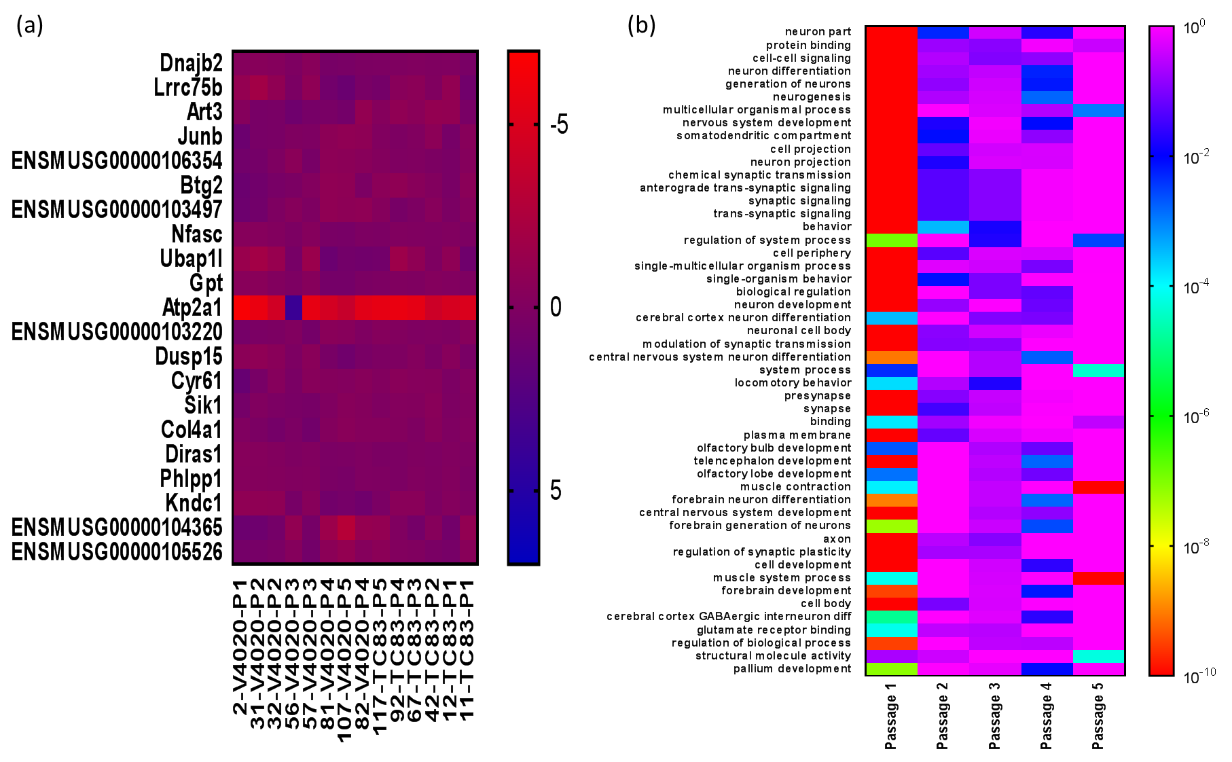


**Figure S4.** RNA-Seq data was generated and trimmed as described in Figure 5. All analysis described was performed using the named modules in the Galaxy web-based platform. Sequences were aligned to the mouse mm10 genome using HiSat2, and features were counted with htseq-count. (**a**) All genes with significant differential expression in a model accounting for both virus vaccine strain and passage number are presented in a heat map showing fold change. All genes with differential expression with an adjusted p-value greater than 0.05 from each individual passage that were detected by DeSEQ2 were used as the input to run GOseq on each passage individually. The pathways reported by GOseq for each passage were combined and sorted by significance. (**b**) The overall top 50 over-represented ontologies are displayed in a heat map depicting the associated adjusted p-value associated with the likelihood of over-representation from GOseq. p-values less than 10^-10^ are reported as 10^-10^.

**Table S1.** Mouse and viral genes that were differentially expressed with an adjusted *p*-value < 0.05 and an absolute value of log2(FC) > 2 from the NanoString nCounter neuro-inflammation array with probes for viral genes added. Fold changes (FC) are reported as the change in V4020 expression compared to TC-83. Adjusted p-values were computed by nSolver using a model to compare individual samples by day of collection.

**Table S2.** Mouse genes that were differentially expressed with an adjusted p-value <0.05 from RNA-Seq on 5 passages of V4020 and TC-83 in mouse brain as reported by DESeq2 model of virus x passage or for comparison of samples in individual passages. FC are reported as the change in V4020 expression compared to TC-83. Adjusted *p*-values are based on the Benjamine-Hochberg correction.

**Table S3.** Concatenation of gene fold change and significance from Table S1 and Table S2 for genes that were present in both NanoString and RNA-Seq analysis.
